# Dynamic ASXL1 Exon Skipping and Alternative Circular Splicing in Single Human Cells

**DOI:** 10.1371/journal.pone.0164085

**Published:** 2016-10-13

**Authors:** Winston Koh, Veronica Gonzalez, Sivaraman Natarajan, Robert Carter, Patrick O. Brown, Charles Gawad

**Affiliations:** 1 Departments of Oncology and Computational Biology, St. Jude Children’s Research Hospital, Memphis, TN, 38105, United States of America; 2 Departments of Bioengineering and Applied Physics, Stanford University, Stanford, CA, 94305, United States of America; 3 Department of Biochemistry and Howard Hughes Medical Institute, Stanford University, Stanford, CA, 94305, United States of America; Iowa State University, UNITED STATES

## Abstract

Circular RNAs comprise a poorly understood new class of noncoding RNA. In this study, we used a combination of targeted deletion, high-resolution splicing detection, and single-cell sequencing to deeply probe ASXL1 circular splicing. We found that efficient circular splicing required the canonical transcriptional start site and inverted AluSx elements. Sequencing-based interrogation of isoforms after ASXL1 overexpression identified promiscuous linear splicing between all exons, with the two most abundant non-canonical linear products skipping the exons that produced the circular isoforms. Single-cell sequencing revealed a strong preference for either the linear or circular ASXL1 isoforms in each cell, and found the predominant exon skipping product is frequently co-expressed with its reciprocal circular isoform. Finally, absolute quantification of ASXL1 isoforms confirmed our findings and suggests that standard methods overestimate circRNA abundance. Taken together, these data reveal a dynamic new view of circRNA genesis, providing additional framework for studying their roles in cellular biology.

## Introduction

Circular RNA (circRNA) isoforms are widely expressed in many organisms, including isoforms that are conserved across species [1–3]. Some overexpressed circRNAs can compete with linear mRNAs for miRNA-binding, leading to phenotypic changes in developing zebrafish embryos [4, 5]. circRNA expression has also been found to undergo dynamic changes during early embryonic development [6]. The recent identification of MBL and Quaking as proteins required for efficient circular but not linear splicing in cell-specific contexts support the possibility that circRNAs have evolved distinct functions [7, 8]. Although these findings suggest important roles for circRNA in cellular biology, we have a limited understanding of how they are formed. The identification of additional factors that decouple the regulation of circular and linear splicing would provide further evidence that circRNA evolved distinct biological functions, as well as tools to study their biological roles.

ASXL1, a gene that encodes a polycomb group protein commonly mutated in myeloid leukemias, encodes a conserved and abundant circRNA (CircBase ID hsa_circ_0001136) [9]. To better understand the genetic requirements for the genesis of circular RNA, we cloned the entire locus encompassing one of the ASXL1 linear isoforms. We then performed deep sequencing of RNA in both bulk samples and single cells to more comprehensively and quantitatively characterize products of ASXL1 pre-mRNA splicing.

## Results

### Identification of Multiple Circular Isoforms of ASXL1 RNA

**Fig 1:**
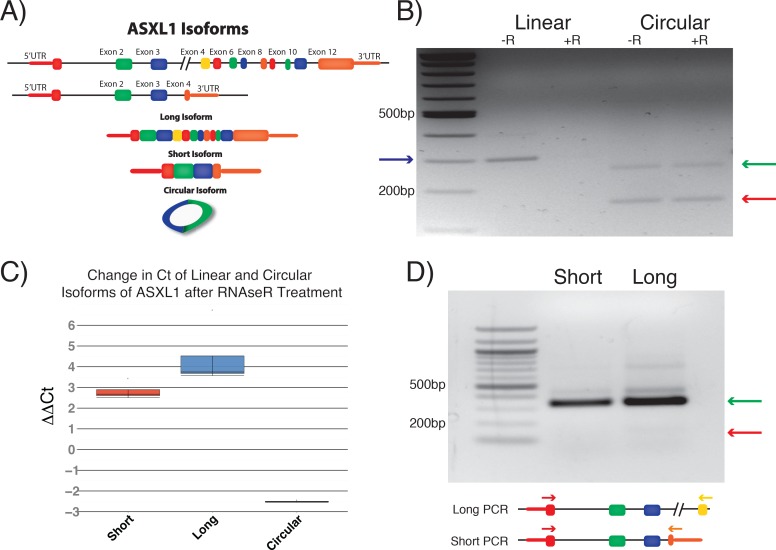
ASXL1 Circular Isoforms. A) Overview of ASXL1 isoforms B) RNAse R resistance assay show degradation of linear isoform (blue arrow) and resistance of the known circular isoform (red arrow). In addition, a second RNAse R resistant isoform was detected in the circular PCR that was confirmed to contain an unannotated exon in intron 3 (exon 3*) by Sanger sequencing C) Quantitation of RNAse R resistance by qPCR showing increased Ct for both linear isoforms after RNAse R treatment but a decreased Ct for the circular isoform D) PCR between exons 1 and 4 for short or long isoforms detect the expected band that corresponds to exons 1 through 4 (green arrow). A band that would have resulted from the removal of exons 2 and 3 through exon skipping was not clearly identified (red arrow).

The ASXL1 locus is transcribed into two distinct linear isoforms, distinguished by the use of an alternative fourth exon (Fig 1A). In addition, there is a circular isoform that is formed between exons 2 and 3, which is conserved between humans and mice [10]. We first confirmed the presence of the ASXL1 circular isoform that is composed of exons 2 and 3 using RNAse R, a riboexonuclease that preferentially digests linear RNA (Fig 1B and 1C). As expected, the predicted circular isoform was resistant to RNAse R while the linear isoform was not. Unexpectedly, there was a second band that was also RNAseR resistant. Sanger sequencing confirmed that it was another circRNA isoform produced by the splicing of exon 3 to an unannotated exon (exon 3*) within intron 3, which was then spliced back to exon 2. To evaluate if the circular isoform was a product of exon skipping, PCR was performed from exons 1 to exon 4 for both the short and long linear isoforms using reverse primers specific to the different fourth exons to identify any products that might have been produced as a result of skipping exons 2 and 3. In both examples, PCR products of the sizes expected for known splice products were detected, but products of splicing between exons 1 and 4 were not clearly identified (Fig 1D).

### ASXL1 Circular RNA is Produced by Splicing from its pre-mRNA

**Fig 2:**
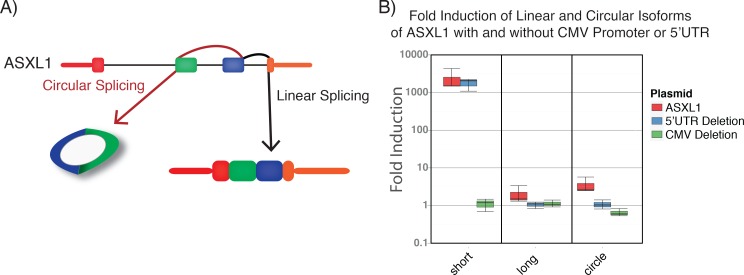
Evaluating Forward versus Back Splicing of ASXL1. A) Schematic showing approach for detecting forward versus back splicing. B) Fold induction of short, long, and circular ASXL1 isoforms after transfection of plasmids with full length gene, plasmid with 5’ UTR removed, or plasmid after deletion of the CMV promoter.

We then cloned the portion of the ASXL1 locus that encodes the short linear RNA isoform and transfected it into HeLa to determine the relative efficiency of splicing exon 3 forward to exon 4 versus splicing back to exon 2 (Fig 2). We were able to detect induction of both the short and circular isoforms of ASXL1 after transfection, with a strong preference of exon 3 splicing forward to exon 4; we estimate linear to circular splicing efficiency to be 500–1000:1. However, the **ΔΔ**Ct values compared to Actin at normal steady state concentrations in HeLa suggested that the circular isoform was present at higher concentrations than the short isoform, consistent with the greater stability of the ASXL1 circular isoform [2], resulting in higher steady-state concentrations as the circular isoform accumulates over time. In addition, we did not detect a change in the concentration of the long ASXL1 isoform after inducing the circular isoform, suggesting there is not a feedback loop of the induced short or circular isoforms to regulate expression or stability of the long isoform.

To determine whether the circular isoform of ASXL1 was spliced from the canonical linear pre-mRNA, or from a transcript initiated at an independent internal transcricriptional start site (TSS) in intron 1 that leaves an orphan 5’ splice site, we deleted most of the CMV promoter from the construct containing the ASXL1 locus. As expected, induction of the short isoform was no longer detected. In addition, we no longer detected induction of the circular isoform. Thus, the circular isoform does not appear to originate from a transcript initiated at an internal circular isoform-specific TSS. We also noted that all three isoforms had slightly lower expression after deletion of the 5’ untranslated region (5’UTR), suggesting that sequence within the 5’UTR could increase transcription of the pre-mRNA that could later be spliced into the long, short, or circular isoforms.

### Inverted Alu Sx is Required for Efficient Circular Splicing

**Fig 3:**
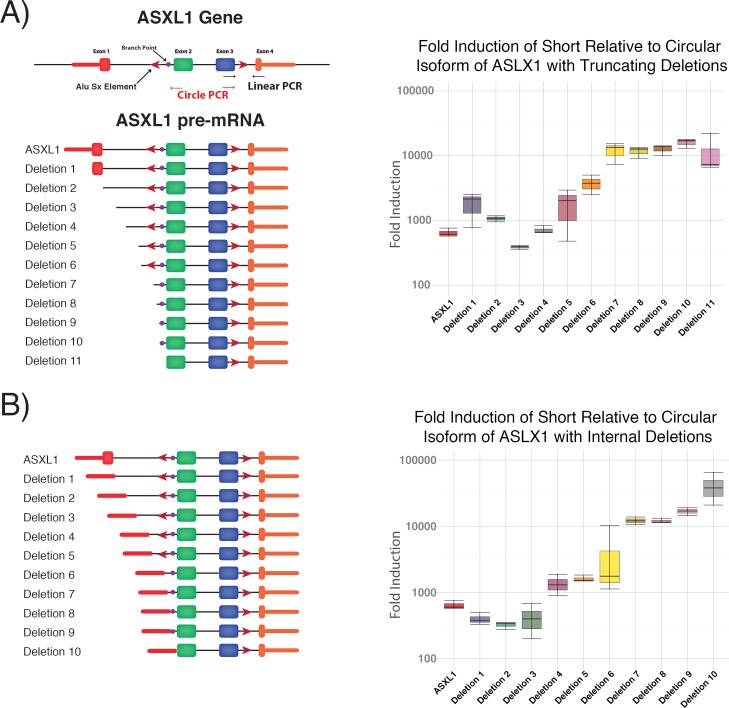
Determination of Sequence Requirements for ASXL1 Circularization. A) Truncating deletions of the ASLX1 gene identify a region (deletion 7) upstream of the branch site that includes an Alu Sx element and produces a significant increase in the ratio of linear to circular RNA formation B) Deletion internal to the 5’UTR identify the same region as an important regulator of preferential linear to circular splicing.

To identify regions that are required for preferential ASXL1 circularization we made a series of truncating deletions of the cloned ASXL1 locus. We chose to make truncations at the end of blocks of highly conserved regions (Fig 3). We then measured the relative formation of the linear to circular isoforms to determine whether any of the tested sequences promoted preferential formation of either isoform. Deletion of the 5’UTR led to a decrease in the levels of both short and circular isoforms, but no change in the relative frequency of circularization consistent with a general decrease in transcription of the pre-mRNA. Deletion of a conserved 241bp region containing an Alu Sx repeat, 426bp upstream of exon 2 (Fig 3A, Deletion 7), resulted in a 10-fold increase in the ratio of the short linear to circular isoform ratio. The presence of an AluSx element, in the opposite orientation in the third intron 131bp downstream of the third exon is consistent with a proposed model that the inverted complementary sequences promote circular splicing [11]. The increase in the relative linear to circular splicing persisted with further truncations, also supporting the importance of that region to maintain the ratio of linear to circular splicing. Unexpectedly, we continued to see splicing at a lower level with the same linear to circular ratio after removing the sequence from the branch point to the 3’ splice site (Deletion 11). We found an AG required for defining of a 3’ splice had been inserted upstream of the new junction after the deletion, as well as pyrimidine-rich stretch (CCGCCGCCGCC) and a sequence 28bp upstream (AGGTCCCGC) that could serve as a branch point. Thus, the deletion inserted a new sequence in front of the exon that likely created a new splice site that allowed for continued splicing but with less efficiency. To further evaluate a potential role for the 5’UTR in RNA circularization, we made a series of deletions internal to the 5’UTR. We did not identify evidence for preferential linear or circular splicing compared to the same deletions where the 5’UTR had been retained. However, we again found that the ratio of the short linear RNA to the circular isoform increased significantly with removal of the region that contained the Alu Sx repeat upstream of the 3’ splice acceptor (Fig 3B, Deletion 6). That increased relative efficiency of linear splicing was observed for all deletions encompassing this Alu Sx element, further supporting a role in efficient circularization. Thus, our data support previous models that inverted repeats can promote formation of circRNAs [2].

### Deep Sequencing Identifies Reciprocal Exon Skipping Products for the Detected Circular Isoforms

**Fig 4:**
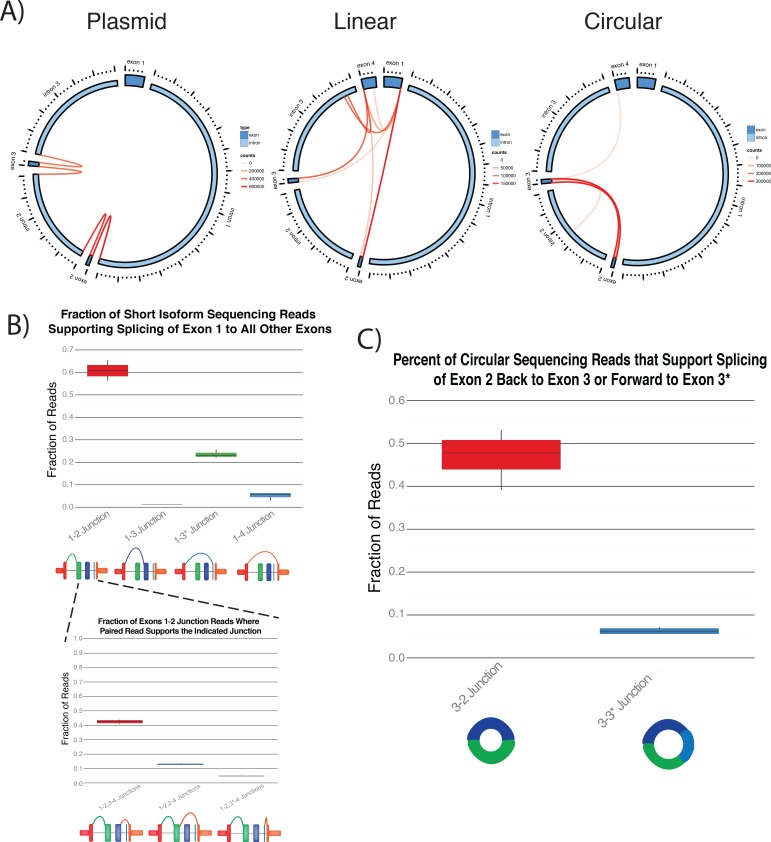
Global Identification of ASXL1 Splice Products after Overexpression of the ASXL1 Locus. A) Exons and introns are depicted around the plot. Locations where a breakpoint was identified are represented by connecting lines, where the color of the line is proportional to the number of reads corresponding to that junction. Unknown junctions were almost exclusively restricted to exon-exon boundaries. Only breakpoints with greater 1% of reads supporting the junction were mapped. B) The preference of exon 1 to splice forward to exon 2, 3, 3*, or 4 were quantitated using primers that amplified from exon 1 to exon 4. The 3’ splicing junction was then quantitated for reads that spliced from exon 1 to 2 on the 5’ end. Most reads corresponded to the expected consecutive junctions, but a significant minority of reads corresponded to non-canonical junctions. C) Quantification of the circular isoforms that either included or excluded exon 3*. Of note, the abundance of the 3–2 circular isoform correlated with the abundance of the reciprocal exon skipping product from exon 1 to exon 3*.

To more globally quantify the diversity of splice products that can form from the ASXL1 locus, we performed PCR for the linear and circular isoforms after overexpression of the plasmid containing the ASXL1 locus, followed by deep sequencing. From the resulting data, we were able to identify the expected splice junctions for both the linear and circular isoforms, including exon 3* within intron 3 in both linear and circular isoforms (Fig 4A). In addition, we unexpectedly found evidence that exons from the linear isoform could less efficiently be spliced to most other exons, resulting in junctions that were not previously known to be contained in mature transcripts. In addition, the putative splice junctions we detected by sequencing were almost exclusively at canonical exon boundaries, suggesting they were not the result of template-switching during reverse transcription, misalignment, or other technical artifacts. As a control for our experimental and computational pipelines, we also sequenced the untransfected plasmid and found only the expected intron-exon junctions, demonstrating that the identified junctions were not an artifact of our analysis pipeline.

Most strikingly, we found promiscuous splicing of exon 1 to all other exons. The most abundant non-canonical products were between exon 1 and the exon 3* in intron 3 (skipping exons 2 and 3), comprising a mean of 23.2% of the amplicon reads. The next most abundant product, comprising 5.7% of reads, joined exons 1 and 4, skipping exons 2, 3, and the novel exon–the constituents of the lower abundance circular isoform. When the second pair of each read was examined to identify the paired 3’ exon junctions, additional non-canonical splicing events were identified in a minority of cases. For example, a median of 12.9% and 4.7% of PCR products with 1–2 exon junction on the 5’ end had 2–4 and 2–3* junctions on the 3’ end, which would skip exon 3 plus the novel junction or exon 3, respectively (Fig 4B). Interestingly, the 3–2 circular isoform was more abundant than the circular isoform that contained the exon 3*, which correlated with the abundance of their reciprocal exon skipping products (Fig 4C). The exon-skipping products are not predicted to encode a protein, suggesting they are not performing distinct functions from their full length linear counterparts and raising the possibility that the exon skipping occurs in a regulated process to produce the circular isoform.

### Heterogeneous Expression of Linear and Circular Isoforms in Single Human Cells

**Fig 5:**
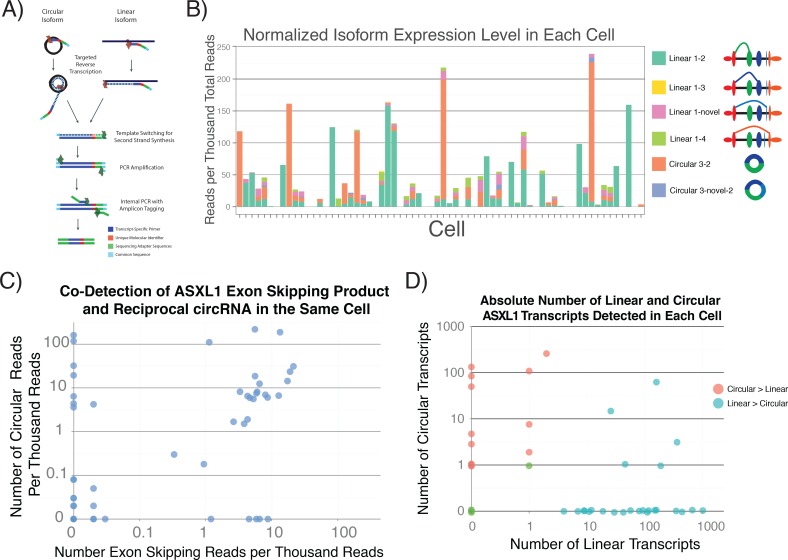
Quantification of ASXL1 Isoforms in Individual Human Cells. 1) Overview of strategy for single cell ASXL1 isoform quantification. Targeted reverse transcription adds a common sequence, Nextera sequences, and a unique molecular identifier. Products then undergo a series of amplifications to produce full length amplicons that are tagged with a UMI. B) Quantification of each of the isoforms in each cell. Either the canonical linear isoform or 3–2 circular isoform comprise the majority of the reads for each cell that expresses ASXL1. Cells also tend to have either high or little to no ASXL1 expression. C) The 1–3* junction reads co-occur in the same cell as the reciprocal circular isoform in contrast to the canonical linear isoform, which is not to be highly expressed in the same cell as the circular isoform. Most cells that only express one of the isoforms express the circular isoform, which may be due to increased stability. D) Absolute quantification of ASXL1 isoforms confirms a preference for expressing either the linear or circular isoform and estimate an average of 11 circular isoform molecules per cell compared to about 64 linear molecules.

Exon skipping has been detected in bulk samples where the reciprocal circRNA was identified [12, 13]. Some have argued that these products are just transcriptional noise with no biological relevance. To determine if variation in circular RNA expression occurs in single cells, we performed targeted single cell ASXL1 linear and circular isoform expression profiling (Fig 5A). Consistent with the bulk data, the canonical linear isoform and 3–2 circular isoform comprised the majority of the sequencing reads. Unexpectedly, in the majority of cases, either the linear isoform or circular isoform constituted the majority of the sequencing reads for that cell. One potential explanation for this finding is that the production of each of the isoforms is mutually exclusive. In addition, the majority of those cells had expression several fold higher than the mean (Fig 3B), consistent with recent single cell reports that transcription occurs in bursts [14, 15].

To search for more direct evidence linking exon skipping to circular splicing, we quantified the exon-skipping linear RNA product in which the first exon is spliced to exon 3* intron 3, the excised intron of which could be a precursor to the 3–2 circRNA. Using this strategy, we found that most cells containing this exon-skipping RNA also contained the reciprocal circular isoform. Interestingly, in these cases, the circular isoform tended to be expressed at a higher level. In some cells we detected the circular isoform but not the corresponding exon-skipping linear product. A possible explanation for these data could be that the circular isoform is more stable than the exon-skipping linear product.

Reliable quantitation of circRNA species is made difficult by the known strand-displacement activity of reverse transcriptases, which could result in rolling circle amplification of circRNA signal during qRT-PCR or sequencing using standard RNA-seq library prep. To estimate the number of isoform molecules in each cell, we used unique molecular identifiers (UMI) during reverse transcription (Fig 5A), and then collapsed molecules with the same identifier prior to quantification[16]. Using this approach, we estimate an average of 64 linear molecules per cell, compared to about 11 circular molecules per cell. In contrast, based on their relative Ct values, the circular ASLX1 molecules are estimated to be more abundant than the linear molecules. Thus, standard qRT-PCR detection methods overestimate circular RNA. Still, based on UMI-tagged sequencing, some cells contained 100 copies of the circular RNA, which would constitute roughly 0.1% exome-derived transcripts from a cell assuming a total of roughly 100,000 messenger RNA molecules per cell. Consistent with the uncorrected data, after UMI quantification, most cells showed almost mutually exclusive expression of the linear and circular isoforms. Thus, although standard methods overestimate circRNA abundance, their expression is heterogeneous, with some cells showing high levels of circRNA expression.

## Discussion

The present study was designed to better understand the origin of circRNAs, which constitute a significant fraction of the human transcriptome but are still poorly understood [17]. We used ASXL1 as a case study, as it encodes an abundant and conserved circRNA. We found evidence for specific sequence elements required for efficient ASXL1 circularization. Our evidence suggested that the circular isoform, like the conventional linear transcript, was spliced from a precursor RNA initiated at the canonical TSS, as has also been recently shown by others [18]. When ASXL1 was overexpressed by transient transfection, the linear RNA isoforms were about five hundred to a thousand-fold more abundant than circular RNA molecules. However, the relatively higher steady-state abundance of circular isoforms, roughly 1/6^th^ that of the linear isoforms, presumably results from the greater stability of circular RNA molecules [2]. We found evidence consistent with a role for flanking, oppositely oriented (hence complementary) Alu sequences, in efficient circularization. This configuration has also been reported in other studies of circular RNA genesis [2, 11, 19].

High-resolution examination of AXL1 splicing revealed many lower frequency linear isoforms, the two most abundant of which skip the exons involved in ASXL1 circularization. Exon skipping has previously been suggested to underlie circRNA formation [12, 20, 21]. If the circular splicing occurs after an alternative splicing event that excludes the circularized exons, they would be processed out of a lariat intermediate. In support of this model, a recent study in *Schizosaccharomyces pombe* found circRNA can be derived from a lariat intermediate [22].

**Fig 6:**
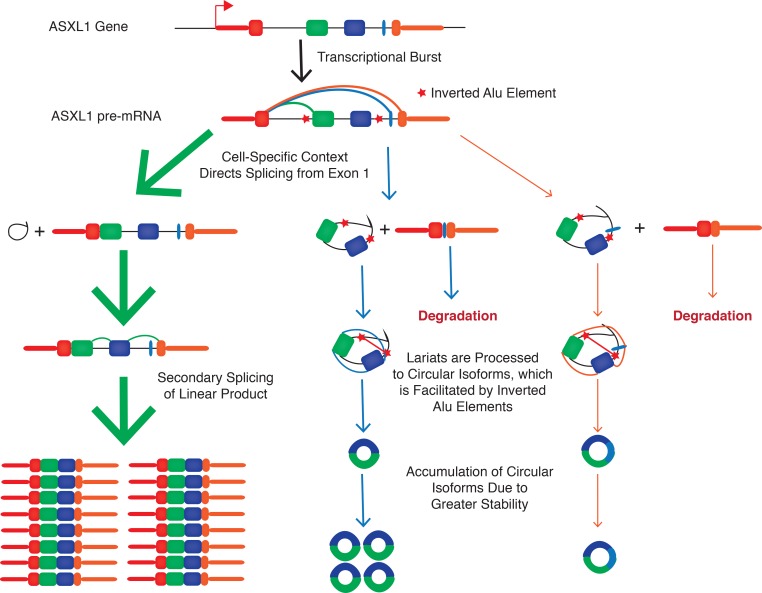
Proposed Model for ASXL1 Circularization. Our single cell data provides evidence that the ASXL1 locus undergoes transcriptional bursts. The resulting transcripts undergo canonical linear splicing or create alternatively spliced products that skip exons 2 and 3, which may occur in a cell context-specific manner. Either the linear or circular products include exon 3* within intron 3 (blue or orange path). The resulting lariats then undergo further processing to create the detected circular isoforms, which is facilitated by the inverted Alu Sx repeats (red arrows). The initial circular splicing occurs at a low level, but the circular isoforms accumulate over time due to their increased stability as a result of exonuclease resistance.

By using single cell sequencing to quantify cell-to-cell variation in isoform levels we found that the circRNA and canonical linear isoforms were co-expressed at high levels in only a minority of cells, but circRNA molecules tended to be found in the same cells with their reciprocal exon-skipping product. Further, we found that all RNA isoforms tended to be present at either high or low levels, consistent with recent studies of single-cell transcription that suggest locus-specific transcription occurs in bursts across the genome which is not detected when the transcriptional programs of cells are averaged out in measurements of mixed populations of cells [14, 15, 23]. Our findings have been summarized in a new model of circular RNA formation (Fig 6).

Although it is still possible that we are providing new details of transcriptional noise through higher resolution interrogation of transcription and splicing, the fixation of the inverted Alu elements required for circularization in the human genome, evidence for the circular isoform being the predominant transcript in some cells, and heterogeneous expression of circular isoforms all lend more weight to the assertion that circRNA are a functional new class of RNA that have evolved regulatory controls and biological functions distinct from those of their linear counterparts. In fact, exon skipping may not be transcriptional noise, but a tightly orchestrated gene expression program that selectively yields circRNA. Further, the tendency for mutually exclusive expression of either linear or circular isoforms in individual cells suggests there may be a regulatory mechanism that directs splicing to occur either between adjacent exons site or between non-adjacent exons, with the skipped exons in the excised lariat serving as precursors in circRNA genesis. These new observations, suggesting potential mechanisms for circRNA formation and its regulation, provide a starting point for further investigations to understand their potential roles in biological regulation.

## Materials and Methods

### RNAse R Treatment, Plasmids, Cell Culture, and Transfection

RNAse R and qPCR experiments were performed as previously described [3]. Plasmids were ordered from companies that use traditional mutagenesis (Mutagenex Suwanee, GA) or a combination of traditional cloning with gene synthesis (Genewiz South Plainfield, NJ). The original plasmid construction required subcloning of fragments prior to ligating the fragments together into the 19.6 kb construct. All plasmids were confirmed using restriction digestion and Sanger sequencing. HeLa Flp-in Trex cells were maintained as previously reported [24]. For each transfection 0.5ug of plasmid were transfected into one well of a 24 well plate that had been seeded with 100,00 HeLa cells 24h prior using using 1ul of dreamfect gold (OZ Biosciences San Diego, CA). Total RNA was isolated 48 hours later using the RNeasy Plus kit (Qiagen Valencia, CA). 400ng of total RNA were then converted to cDNA using Superscript VILO (Thermo Fisher Scientific Waltham, MA).

### qPCR

cDNA from 20ng of total RNA were used in qPCR reactions using 20X Taqman Master Mix (Thermo Fisher Scientific Waltham, MA) and 1X qPCR assay (*Integrated DNA Technologies*, *Coralville*, *IA*). qPCR was run on a Stratagene MX3005P using standard 2 cycle protocol. Ct values were determined using thresholds that were automatically determined by the Stratagene software for each assay. Each ASXL1 isoform was normalized to beta actin, and a GFP was used as a negative experimental control. All assays were done with three biological replicates and standard box plot values were used to visualize the data (line representing median, first and third quartiles also know as the interquartile range represented by the box, and whiskers representing the smallest observation greater than or equal to either end of the interquartile range +/- 1.5 * interquartile range.

qPCR Assays (all from Integrated DNA Technologies, Coralville, IA)

Actin

Predesigned Assay Hs.PT.56a.19461448.g

Short

F GTT TTA TAA ACT GCC TGG CCG

R GAC TAA CAG ACC ACT CCC AAG

Probe /56-FAM/TCA GCC TTT /ZEN/TCA CGC TCA AGG TGT /3IABkFQ/

Long

F CAG CAT CAC CCC AGT CTT TT

R CGA CCT CTT TCC AAT CCC AG

Probe /56-FAM/AGA GCT TCC /ZEN/TCA CAG GCG AAC AAA /3IABkFQ/

Circle

F GAA TCA GCC TTT TCA CGC TC

R TCC TTC TGC CTC TAT GAC CTG

Probe /56-FAM/ACT CGG ATG/ZEN/CTC CAA TGA CAC C /3IABkFQ/

### Deep Sequencing of PCR Products

We then performed PCR with cDNA made from 10ng of total RNA using primers specific for the linear or circular isoforms using the FastStart High Fidelity PCR System according to the manufacturer’s instructions (Roche Life Sciences Branford, CT). In addition, primers were used to amplify exon 2 or exon 3 from the plasmid to as a control for our experimental and informatics pipelines. The common tag TCGTCGGCAGCGTCAGATGTGTATAAGAGACAG was added to all forward primers while GTCTCGTGGGCTCGGAGATGTGTATAAGAGACAG was added to the reverse primers. The product was diluted 1:100 and standard Nextera indexing primers were then used in a second PCR to add adapters and indices (Illumina), again using the FastStart High Fidelity PCR System (Roche Life Sciences Branford, CT). The amplicons from all experiments were pooled and sequenced on a single MiSeq lane with 2X150bp reads using v2 chemistry (Illumina San Diego, CA). These experiments were performed with four biological replicates and standard box plots were computed as described above.

Amplicon Primers (common sequences added when samples were prepared for sequencing)

Long F TGCTCCAATGACACCAAAAC

Long R AGACCCACAGCTCTCCACAT

Short F GGGAGAAGGATGAAGGACAA

Short R CCCAAGCTTACAGCAGGTTC

Circle F TCAATGCTATGCTACATTCCAATTC

Circle R ATTGAGGCATGCGAGAGG

Plasmid Primers

exon 2 F CCAGCGGTACCTCATAGCAT

exon 2 R TGAAACCCTCATGTTAAGCAA

exon 3 F TGGATTGTATAACCCTCATCCA

exon 3 R AAATTCATGGCCCCTATTCC

Analysis of Bulk Samples

Reads were demultiplexed using Illumina software, followed by isolation of reads specific to each PCR reaction using grep to identify reads containing specific primer sequences. For determining splice junctions, reads were clustered using DNAclust (http://dnaclust.sourceforge.net/) using–s 0.99 and–k 10 options. We required at least 5 reads be present in each cluster and then aligned the clusters to the ASXL1 reference introns and exons using mummer (http://mummer.sourceforge.net/) with options (-l 18 -c 25 -p) where the location and count for alignments were retained. To quantify the canonical splicing and 1–4 exon skipping products, reads that matched tens bases on each side of the respective junctions were counted while allowing for a single mismatch. Results for insertion counts and splicing junctions were then visualized using R with ggbio (http://www.bioconductor.org/packages/release/bioc/html/ggbio.html) and ggplot2 (http://ggplot2.org/).

### Single Cell Transcript Quantification

Single HeLa cells were grown to 70–80 percent confluence prior to tryspsinazation. Cells were collected in PBS, washed twice in C1 wash buffer, strained with 20 micron Pluriselect filters (San Diego, CA), and loaded on C1 according to manufacturer’s instructions (Fluidigm, South San Francsico, CA). Custom scripts (available at https://www.fluidigm.com/c1openapp) using Clontech Smart-Seq V4 reatents (Mountain View, CA) with custom primers replacing the standard oligo were used during reverse transcription at 12μM. The UMI was immediately upstream of the priming sequencing, followed by a filler sequence, Nextera Read 2 sequences, and Clontech PCR Primer IIA sequence. After amplification, the samples were diluted followed by a second PCR reaction using the short and circular-specific PCR primers with i7 Nextera adapter sequences (Illumina San Diego, CA) using the PCR protocol described above. A second dilution and PCR with Nextera i5 and i7 adapters was performed, followed by pooling and Ampure Bead (Beckman Coulter Indianapolis, IN) cleanup using a 1:1.8 ratio. The library was run on an Illumina MiSeq using V2 chemistry (San Diego, CA). All chambers with a single cell visualized by light microscopy were included in the analyses.

Short_sc_UMI RT Primer

AAGCAGTGGTATCAACGCAGA(Clontech_PCR_Primer_IIA)GTGTCTCGTGGGCTCGGAGATGTGTATAAGAGACAG(Nextera_Read_2_Sequence)TCGA(filler_sequence)NNNNNNNN(UMI)CCCAAGCTTACAGCAGGTTC(linear-specific primer reverse primer)

Circle_sc_UMI RT Primer

AAGCAGTGGTATCAACGCAGA(Clontech_PCR_Primer_IIA)GTGTCTCGTGGGCTCGGAGATGTGTATAAGAGACAG(Nextera_Read_2_Sequence)AGCT(filler_sequence)NNNNNNNN(UMI)ATTGAGGCATGCGAGAGG(circle-specific reverse primer)

Short_sc_Forward Primer

TCGTCGGCAGCGTCAGATGTGTATAAGAGACAG(Nextera_Read_1_Sequence)ATGAAGGACAAACAGAAGAAGAAGA(linear-specific forward primer)

Circle_sc_UMI Forward Primer

TCGTCGGCAGCGTCAGATGTGTATAAGAGACAG(Nextera_Read_1_Sequence)TCAATGCTATGCTACATTCCAATTC(circle-specific forward primer)

### Analysis of Single Cell ASXL1 Isoform Expression

Reads were again demultiplexed using Illumina software (San Diego, CA), followed by removal of artifacts from the multiplexed RT that contained a linear primer on one end and a circular on opposite end prior to quantification of total reads per cell. The files were then separated into linear and circular reads using the RT primer sequences in read 2. Seqtk (https://github.com/lh3/seqtk) was then used to extract paired reads for the linear or circular reads, which was followed by counting of reads that contained each of the junctions using ten base pairs on either side of the junction and allowing for a single mismatch. Linear and circular reads underwent absolute quantification by isolating reads that contained the linear or circular RT primer sequence and a linear or circular junction, respectively, followed by UMI isolation, counting, and retaining of counts that had at least 2 reads that were perfectly matched.

## Supporting Information

S1 Table Source Data for Fig 5. Raw and normalized read, junction, and UMI count data for each cell included in Fig 5.
(XLSX)

## References

[1] Salzman J, Chen RE, Olsen MN, Wang PL, Brown PO. Cell-type specific features of circular RNA expression. PLoS genetics. 2013;9(9):e1003777 10.1371/journal.pgen.1003777 24039610 PMC3764148

[2] Jeck WR, Sorrentino JA, Wang K, Slevin MK, Burd CE, Liu J, et al Circular RNAs are abundant, conserved, and associated with ALU repeats. Rna. 2013;19(2):141–57. 10.1261/rna.035667.112 23249747 PMC3543092

[3] Salzman J, Gawad C, Wang PL, Lacayo N, Brown PO. Circular RNAs are the predominant transcript isoform from hundreds of human genes in diverse cell types. PloS one. 2012;7(2):e30733 10.1371/journal.pone.0030733 22319583 PMC3270023

[4] Memczak S, Jens M, Elefsinioti A, Torti F, Krueger J, Rybak A, et al Circular RNAs are a large class of animal RNAs with regulatory potency. Nature. 2013;495(7441):333–8. 10.1038/nature11928 . 23446348

[5] Hansen TB, Jensen TI, Clausen BH, Bramsen JB, Finsen B, Damgaard CK, et al Natural RNA circles function as efficient microRNA sponges. Nature. 2013;495(7441):384–8. 10.1038/nature11993 . 23446346

[6] Fan X, Zhang X, Wu X, Guo H, Hu Y, Tang F, et al Single-cell RNA-seq transcriptome analysis of linear and circular RNAs in mouse preimplantation embryos. Genome biology. 2015;16:148 10.1186/s13059-015-0706-1 26201400 PMC4511241

[7] Ashwal-Fluss R, Meyer M, Pamudurti NR, Ivanov A, Bartok O, Hanan M, et al circRNA Biogenesis Competes with Pre-mRNA Splicing. Molecular cell. 2014 10.1016/j.molcel.2014.08.019 . 25242144

[8] Conn SJ, Pillman KA, Toubia J, Conn VM, Salmanidis M, Phillips CA, et al The RNA Binding Protein Quaking Regulates Formation of circRNAs. Cell. 2015;160(6):1125–34. 10.1016/j.cell.2015.02.014 . 25768908

[9] Gelsi-Boyer V, Trouplin V, Adelaide J, Bonansea J, Cervera N, Carbuccia N, et al Mutations of polycomb-associated gene ASXL1 in myelodysplastic syndromes and chronic myelomonocytic leukaemia. Br J Haematol. 2009;145(6):788–800. 10.1111/j.1365-2141.2009.07697.x . 19388938

[10] Wang PL, Bao Y, Yee MC, Barrett SP, Hogan GJ, Olsen MN, et al Circular RNA is expressed across the eukaryotic tree of life. PloS one. 2014;9(6):e90859 10.1371/journal.pone.0090859 24609083 PMC3946582

[11] Capel B, Swain A, Nicolis S, Hacker A, Walter M, Koopman P, et al Circular transcripts of the testis-determining gene Sry in adult mouse testis. Cell. 1993;73(5):1019–30. Epub 1993/06/04. . 7684656 10.1016/0092-8674(93)90279-y

[12] Zaphiropoulos PG. Exon skipping and circular RNA formation in transcripts of the human cytochrome P-450 2C18 gene in epidermis and of the rat androgen binding protein gene in testis. Molecular and cellular biology. 1997;17(6):2985–93. Epub 1997/06/01. 9154796 10.1128/mcb.17.6.2985 PMC232150

[13] Kelly S, Greenman C, Cook PR, Papantonis A. Exon Skipping Is Correlated with Exon Circularization. J Mol Biol. 2015;427(15):2414–7. 10.1016/j.jmb.2015.02.018 . 25728652

[14] Deng Q, Ramskold D, Reinius B, Sandberg R. Single-cell RNA-seq reveals dynamic, random monoallelic gene expression in mammalian cells. Science. 2014;343(6167):193–6. 10.1126/science.1245316 . 24408435

[15] Dar RD, Razooky BS, Singh A, Trimeloni TV, McCollum JM, Cox CD, et al Transcriptional burst frequency and burst size are equally modulated across the human genome. Proceedings of the National Academy of Sciences of the United States of America. 2012;109(43):17454–9. 10.1073/pnas.1213530109 23064634 PMC3491463

[16] Kivioja T, Vaharautio A, Karlsson K, Bonke M, Enge M, Linnarsson S, et al Counting absolute numbers of molecules using unique molecular identifiers. Nature methods. 2012;9(1):72–4. 10.1038/nmeth.1778 . 22101854

[17] Jeck WR, Sharpless NE. Detecting and characterizing circular RNAs. Nature biotechnology. 2014;32(5):453–61. 10.1038/nbt.2890 24811520 PMC4121655

[18] Ashwal-Fluss R, Meyer M, Pamudurti NR, Ivanov A, Bartok O, Hanan M, et al circRNA biogenesis competes with pre-mRNA splicing. Molecular cell. 2014;56(1):55–66. 10.1016/j.molcel.2014.08.019 . 25242144

[19] Liang D, Wilusz JE. Short intronic repeat sequences facilitate circular RNA production. Genes & development. 2014;28(20):2233–47. 10.1101/gad.251926.114 25281217 PMC4201285

[20] Kelly S, Greenman C, Cook PR, Papantonis A. Exon Skipping Is Correlated with Exon Circularization. Journal of molecular biology. 2015 10.1016/j.jmb.2015.02.018 . 25728652

[21] Gualandi F, Trabanelli C, Rimessi P, Calzolari E, Toffolatti L, Patarnello T, et al Multiple exon skipping and RNA circularisation contribute to the severe phenotypic expression of exon 5 dystrophin deletion. Journal of medical genetics. 2003;40(8):e100 Epub 2003/08/16. 10.1136/jmg.40.8.e100 12920092 PMC1735543

[22] Barrett SP, Wang PL, Salzman J. Circular RNA biogenesis can proceed through an exon-containing lariat precursor. eLife. 2015;4:e07540 10.7554/eLife.07540 26057830 PMC4479058

[23] Shalek AK, Satija R, Adiconis X, Gertner RS, Gaublomme JT, Raychowdhury R, et al Single-cell transcriptomics reveals bimodality in expression and splicing in immune cells. Nature. 2013;498(7453):236–40. 10.1038/nature12172 23685454 PMC3683364

[24] Gassmann R, Holland AJ, Varma D, Wan X, Civril F, Cleveland DW, et al Removal of Spindly from microtubule-attached kinetochores controls spindle checkpoint silencing in human cells. Genes & development. 2010;24(9):957–71. 10.1101/gad.1886810 20439434 PMC2861194

